# Sustained attention and inhibitory control: age and sex related difference in children and adolescents using a CPT with distracting events

**DOI:** 10.3389/fpsyg.2025.1609537

**Published:** 2025-08-20

**Authors:** Carlotta Rivella, Paola Viterbori, Maria Carmen Usai

**Affiliations:** Department of Education Sciences, University of Genoa, Genoa, Italy

**Keywords:** sustained attention, inhibitory control, MOXO d-CPT, age and sex differences, distractors

## Abstract

**Introduction:**

The study investigates age and sex-related differences in sustained attention and inhibitory control in a sample of children and adolescents using a continuous performance test with distractor events. In addition, the impact of distractors on sustained attention and inhibitory control is explored.

**Methods:**

The study included 479 individuals aged 6–17 years and analyzed four indices, namely omission, timing, impulsivity, and hyperactivity.

**Results:**

Results revealed that both sustained attention and hyperactivity show age-related changes into adolescence, whereas impulsivity shows age-related changes only in the 6–12 age range, with no differences observed from 13 to 17. Sex differences emerged in inhibitory control: impulsivity remained consistently lower in females than in males across the entire 6–17 age range. In contrast, sex differences in hyperactivity were no longer evident by age 17. Overall, combined distractors have the greatest negative impact on performance, followed by visual and auditory distractors. However, in adolescents, lower distractors impact emerged, together with a positive impact of the auditory ones.

**Discussion:**

These findings provide helpful insight on sustained attention an inhibitory control development, showing different trajectories for impulsivity and hyperactivity. In addition, insight on the role of distractors in determining the performances emerged.

## Introduction

This study investigates age- and sex-related differences in sustained attention and inhibitory control in a sample of children and adolescents aged 6–17, using a continuous performance test (CPT) incorporating distractor events. Additionally, it inspects the impact of these distractors on performance, considering individual differences in age and sex.

Sustained attention, inhibitory control, impulsivity, and hyperactivity are interrelated cognitive and behavioral constructs that often co-occur and influence one another, particularly in the context of self-regulation (Barkley, 1997; Petersen and Posner, 2012). Sustained attention is the ability to maintain the attentional focus over time on a given task without being distracted (Betts et al., 2006). This ability is crucial in daily life as it is essential in tasks that require continuous monitoring or concentration, such as work-related or school-related tasks (e.g., reading, driving, or studying; Petersen and Posner, 2012). Inhibitory control—a core executive function enabling individuals to suppress automatic or irrelevant responses—supports sustained attention by filtering distractions and maintaining task-relevant focus. Deficits in inhibitory control can lead to impulsive behavior and difficulty maintaining attention over time. For example, individuals with poor inhibition may struggle to resist distractions or interrupt ongoing tasks with unrelated actions or thoughts, thereby reducing sustained attention. Similarly, hyperactivity—characterized by excessive motor activity—may reflect an inability to inhibit movement, further disrupting attentional focus. Inattention and hyperactivity were found highly related to one another within a sample of children and young people with problems in attention, learning and memory reported by specialized operators thus the inattention and hyperactivity symptoms may be considered as transdiagnostic symptom dimensions (Zdorovtsova et al., 2023).

The development of attention and inhibitory control is a prolonged and dynamic process involving the maturation and integration of distributed neural systems. Attention, as conceptualized by Petersen and Posner (2012), comprises three distinct but interconnected components—alerting, orienting, and executive control—each relying on partially distinct neural circuits: the alerting network involves the locus coeruleus and right frontal/parietal regions; the orienting system is centered in the superior parietal lobule and frontal eye fields; while executive control predominantly recruits the anterior cingulate cortex and dorsolateral prefrontal cortex. These systems cooperate to support goal-directed behavior and selective information processing. Although sustained attention and inhibitory control are distinct cognitive functions, they are closely interconnected as they rely on overlapping neural mechanisms, notably within frontostriatal and frontoparietal networks that involve the prefrontal cortex, including the dorsolateral prefrontal cortex and anterior cingulate cortex (Corbetta and Shulman, 2002; Castellanos and Proal, 2012).

Sustained attention emerges in early childhood and continues to refine through adolescence, in parallel with the functional maturation of the prefrontal cortex (Betts et al., 2006; Ordaz et al., 2013). Its development is tightly linked to inhibitory control. Inhibitory control itself is multifaceted, including response inhibition, interference control, and motor inhibition (Diamond, 2013; Kang et al., 2022). Developmentally, inhibitory control begins to emerge within the first year of life and strengthens markedly during early and middle childhood, achieving near-adult levels only during adolescence (Cragg, 2016; Kang et al., 2022). Motor inhibition shows early improvement, while interference control and error monitoring mature later and are supported by increasing functional integration within fronto-striatal and cingulo-opercular networks (Ordaz et al., 2013; Marek et al., 2015). Interestingly, studies show that younger children tend to recruit more diffuse and posterior networks, whereas older children and adolescents progressively shift toward more focal prefrontal engagement, reflecting a neurodevelopmental trajectory from general to specialized processing (Bunge et al., 2002; Durston et al., 2006).

### Age and sex-related differences in sustained attention and inhibitory control using continuous performance tests

The CPT is one of the most widely used paradigms for examining sustained attention and inhibitory control within a single task. It involves the presentation of a series of visual or auditory stimuli over a defined period, during which participants are required to respond to target stimuli and withhold responses to non-target stimuli. Sustained attention is typically assessed through the number of correct responses to target stimuli (accuracy), or conversely, through the number of missed targets (omission errors). Inhibitory control and impulsivity, on the other hand, are evaluated based on the number of commission errors, that is, responses made to non-target stimuli. Greenberg and Waldmant (1993), using age-normed data from the Test of Variables of Attention, reported that performance on sustained attention tasks shows rapid improvement in early childhood, followed by a plateau in adolescence. While these norms reflect cross-sectional performance differences across ages, they provide indirect evidence of a non-linear developmental trajectory in sustained attention between ages 6 and 16. Several more recent studies have supported these results, showing that, regardless of the CPT task used, there is a continuous maturation of sustained attention throughout childhood and adolescence with a period of accelerated development in middle and later childhood, but with the magnitude of gains reducing from around 10 years (e.g., Betts et al., 2006; Conners et al., 2003).

As for inhibitory control, many studies show age-relate changes similar to those observed in sustained attention, with a rapid decrease in commission errors until the age of 6 (e.g., Klenberg et al., 2001) and a plateau of these abilities reached together with the attention one (Conners et al., 2003; Kanaka et al., 2008). However, others found different trajectories. For example, Rebok et al. (1997) found that, after the age of 10, attention shows the greatest gain in development compared to inhibitory control. In a study by Betts et al. (2006), attentional performance improved notably between early and middle childhood, with 8–9-year-olds showing better attention indices than 5–6-year-olds and performing at a comparable level to 11–12-year-olds. However, inhibitory control continued to develop beyond this stage, as significant differences were still evident between the 8–9 and 11–12-year-old groups. Therefore, literature shows both linear and non-linear age-related variations in sustained attention and inhibitory control. Taken together, these findings suggest that age-related changes in sustained attention and inhibitory control, as measured by CPT tasks, may follow a predominantly non-linear developmental trajectory, with periods of rapid improvement followed by a plateau. However, further research is needed to clarify the consistency and nature of these patterns across different age groups and individual differences, including potential sex-related dissimilarities in the development of attentional and inhibitory skills.

Indeed, this represents another open question, namely whether sex-related dissimilarities exist in the developmental trajectories of attentional and inhibitory skills. Although in some studies sex differences emerge, with females showing lower levels of commission errors, some contrasting results exist (see Usai, 2022 for a review). Studies using CPTs have reported no sex differences in school-aged children (Brocki and Bohlin, 2004). Moreover, some discrepancies are found when different indices are considered within the same task. Conners et al. (2003) examined CPT normative data for 816 children aged 9–17 years and reported more inattention (omission error) and more impulsive errors for boys. Cornblath et al. (2019) found that males showed higher impulsivity than females, but no difference in the true positive rate of CPT, which is considered a measure of sustained attention. A meta-analysis of eight studies using CPT in children and adolescents with attention deficit hyperactivity disorder (ADHD) showed higher commission errors in boys than in girls, but no differences in attention (Hasson and Fine, 2012). These results suggest that sustained attention and inhibitory control measures may be differently affected by individual differences due to sex, with sustained attention showing more homogeneous performances between males and females. In contrast, males perform lower than females when measures of inhibitory control are considered. These results are consistent with sex-related differences in brain dynamics observed, for example, in preschool-aged children (Toffoli et al., 2024). Available data suggest that males may exhibit a later maturation of inhibitory control compared to females, whereas no significant sex differences have been consistently observed in the development of sustained attention.

### The present study

Laboratory tests are often criticized for their limited ecological validity, as they typically fail to capture the complexity and unpredictability of real-world environments. As Barkley and Murphy (2010) noted, such tasks may not adequately reflect the daily challenges that demand sustained attention and inhibitory control. In particular, traditional paradigms often lack sudden events or distractors that can interfere with these executive functions. To address this limitation, we employed the MOXO d-CPT, a standardized computerized CPT with embedded distractors, aiming to evaluate the impact of such interference on performance and to explore potential age- and sex-related differences in response to these challenges.

MOXO d-CPT measures the ability to respond accurately to targets independently to how fast the response is (Omissions), respond to targets rapidly during the time of presentation of the stimulus (Timing), inhibit responses to non-targets (Impulsivity), and control motor activity (Hyperactivity). Compared to traditional CPTs, the MOXO includes ecologically valid visual and auditory distractors, increasing task demands. In addition, it provides four different measures: two for sustained attention and two for inhibitory control. Including a void period (that is a period without stimulus on the screen) after each stimulus and using variable presentation durations of the elements, the MOXO-CPT allows for the distinction between accurate responses made with appropriate timing—quick and correct reactions to targets during the stimulus presentation—and accurate but delayed responses, in which correct answers occur after the stimulus presentation, during the subsequent void period. The Timing index includes only on-time responses to target stimuli, while Omission index includes both on-time and delayed responses. This distinction addresses a common limitation of traditional CPTs—namely, the risk of misclassifying a child as inattentive when omissions are actually due to slow response speed rather than deficits in sustained attention or inhibitory control (Conners et al., 2003). It is important to note that correct responses made outside the expected time window are still counted as correct responses, but they also contribute to the omissions score, potentially impacting its interpretation. As for inhibitory control indices, the Impulsivity index considers impulsive behavior only as responses to non-target stimuli as if they were target stimuli—specifically, pressing the spacebar on the keyboard in response to non-target stimuli. The Hyperactivity index, on the other hand, includes all types of commission errors that are not classified as impulsive responses, such as the subsequent of multiple responses to the same stimulus (target or non-target) or pressing any key on the keyboard other than the spacebar. Compared to classic CPT, where commission errors are coded in any case of inappropriate response to the target, having two separate indices allow to differentiate commission errors due to impulsive behavior (respond to a non-target stimulus) from commission errors due to motor control difficulties (Berger et al., 2017). This is in line with the research for characterization and assessment of the motor function in different neurological conditions, including ADHD (Gaddis et al., 2015; Londral et al., 2016). Other studies have already used this task to investigate the performance differences between ADHD group and typically developing children (Berger et al., 2013; Rivella et al., 2024; Slobodin et al., 2018). Berger et al. (2013) found that children with ADHD aged 6–11 exhibited a maturational delay in attention functions, while inhibitory control followed a different developmental trajectory compared to controls. Similarly, Slobodin et al. (2018), in a study comparing sustained attention performance (omission errors) of children and adolescents aged 6–18 with and without ADHD, found that deficits in sustained attention in the ADHD group could be attributed to a maturational delay that improves with age. Additionally, when examining the impact of distractors on performance, they observed that distractibility decreased in non-ADHD adolescents, whereas ADHD participants remained sensitive to distractors even into late adolescence. These findings further support the idea of a distinct developmental trajectory for inhibitory control in children and adolescents with ADHD compared to controls. More recently, Rivella et al. (2024) investigated the impact of distractors on sustained attention and inhibitory control in children aged 8–12 with and without ADHD considering the four MOXO indices. Results indicated that children with ADHD and controls differed in their reaction to distracting stimuli, as visual distractors cause a higher decrease in sustained attention and inhibitory control in the ADHD group.

To summarize, the existing studies investigate age-related differences in sustained attention (Slobodin et al., 2018) and inhibitory control (Berger et al., 2013; Rivella et al., 2024), focusing primarily on children with ADHD, while sex-related differences were not investigated. Moreover, no studies have examined the impact of distractors on inhibitory control or response timing in a sample of adolescents older than 12, nor in relation to sex differences. Addressing this gap is crucial, because adolescence is characterized by a neural mismatch—maturational asynchrony between affective and cognitive systems—that makes inhibitory control particularly susceptible to distraction. Functional and ERP evidence shows that emotionally-salient distractors prolong reaction times and modulate neural markers (N2/P3) more in early adolescence, and that females may exhibit greater latency increases than males when facing emotional distractors. Additionally, sex-specific developmental trajectories in cortico-subcortical inhibitory networks have been documented during adolescence (Chung et al., 2020), suggesting that distractor sensitivity could interact with these maturational differences. Investigating how distractors affect inhibitory control and timing—especially in older adolescents and across sexes—not only advances our theoretical understanding of executive function development but also informs-tailored interventions and educational strategies during a developmental period of heightened vulnerability.

The present study examines age- and sex-related differences in sustained attention and inhibitory control in children and adolescents aged 6–17. Additionally, it investigates the relationship between sustained attention, impulsivity, and hyperactivity, as well as the impact of distractors on these domains. Participants completed the MOXO d-CPT task, which provides four key measures: omissions (Omissions), in-time correct responses (Timing), hyperactive responses (Hyperactivity), and commissions (Impulsivity). The relationships among these measures and their common components were analyzed to determine whether they reflect distinct abilities such as sustained attention and inhibitory control.

Based on prior research (Lewis et al., 2017; Yan et al., 2018), we hypothesized that sustained attention and inhibitory control performance would develop with age in a non-linear trajectory, peaking in early childhood and continuing to improve into late adolescence, regardless of sex. However, due to conflicting findings in the literature, we refrained from speculating whether the developmental peak for these abilities occurs at the same age or at different times. Furthermore, we hypothesized that sex-related differences in impulsivity and hyperactivity would emerge when performing a demanding task like the MOXO d-CPT, with females demonstrating better performance across both age groups (Hasson and Fine, 2012). Furthermore, we predict that visual distractors, alone or combined with auditory distractors, will have a greater impact on MOXO d-CPT performance than auditory distractors, as they share the same cognitive modality as the main task (Wickens, 2008). We also expect a greater impact of distractors in males, with this impact diminishing with age (Rivella et al., 2024).

## Materials and methods

### Participants

An initial sample of 616 individuals (326 females) between 6 and 17 years of age was recruited through public primary and secondary schools located in an Italian northern town. The study was approved by the Ethical Committees for research (CER) of the Department of Education Sciences (CER approval no. 029_1 dated 17/10/2019). Written informed consent was obtained from all parents.

The following inclusion criteria were considered: (i) the participants did not have to have a diagnosis of ADHDs or other neurodevelopmental disorders (i.e., Autism Spectrum Disorder or intellectual disabilities) as reported by parents or themselves in informed consent; and (ii) they should not have been at risk for ADHD according to the ADHD rating scale-5 for children and adolescents (Italian adaptation by Marzocchi and Cavallero, 2019). After verifying these requirements, 58 participants were excluded because they had a certification for learning disability (*n* = 18), ADHD (*n* = 16), comorbidity between specific learning disorders and ADHD (*n* = 4), language disorder (*n* = 3), or a disorder not specified (*n* = 17). Eighty-one participants were also excluded because the screening for the presence of an ADHD risk was positive (*n* = 56) or because they did not return the relevant questionnaires (*n* = 25). A child for whom consent and questionnaires were completed was not tested for repeated absences. Finally, values above or below 3 standard deviations from the mean for each age group in the MOXO d-CPT indices are considered outliers and were removed from the dataset: 32 participants with outlier responses were excluded from the final sample.

Then MOXO d-CPT was administered to 479 individuals (*M*_*age*_ = 10.95, SD = 3.47; see Table 1 for a description). The Kids version was performed by 321 children (159 girls), while the Teens & Adult version was performed by 158 adolescents (102 girls). Information about parent education was obtained for 432 mothers and 414 fathers at five levels (primary, 1st grade secondary, upper secondary, university, and post-graduate): most parents have obtained at least an upper secondary school diploma (80% of mothers and 72% of fathers).

**TABLE 1:** Descriptives for participants characteristics (age, sex, and parents’ education) and MOXO d-CPT indices.

Descriptives	Frequency	Mean (SD)	Median	Skewness–Kurtosis
Age	479	10.95 (3.47)	11	–
Sex F/M	261/218	–	–	–
Mother education	432	3.21 (0.87)	3	–
Father education	414	3 (0.82)	3	–
Omissions	479	14.75 (21.03)	7	3.04–11.08
Timing	479	193.01 (39.22)	201	−0.77 to 0.14
Hyperactivity	479	19.99 (33.55)	8	4.20–24.93
Impulsivity	479	11.49 (8.97)	9	2.15–7.46

SD, standard deviation.

### Procedure

All participants were individually assessed at school using the Moxo- Kids or Teens & Adult version. The task was administered by trained graduate students. Parents were asked to complete a screener questionnaire to assess ADHD symptoms in their children. The MOXO d-CPT includes two task versions tailored to different age groups: one for children aged 6–12 years and another for adolescents and adults aged 13 and older. Consequently, age- and sex-related differences were analyzed separately for these two age groups (6–12 and ≥13 years) across all MOXO d-CPT indicators.

### Measures

#### ADHD symptoms

The *ADHD Rating scale-5 for children and adolescents* (DuPaul et al., 2016; Italian adaptation by Marzocchi and Cavallero, 2019) was administered to parents of children aged 6–17 years. It consists of 18 items that correspond to the 18 ADHD symptoms contained in the DSM-V. Three symptom scores are derived: Inattention, Hyperactivity/Impulsivity, and Total score. For each of the three scores, a higher score corresponds to greater difficulties.

#### MOXO d-CPT

The task was administered to all participants. It consists of eight blocks, each with 53 trials (33 target) for the Kids version and 59 trials for the Teens & Adults version (34 target). In each trial, a stimulus (target or non-target) is presented in the middle of the computer screen for a variable duration of presentation. It is followed by a “void” period of the same duration, during which no stimuli are presented. Participants are requested to respond to the target stimulus as quickly as possible by pressing the spacebar once and to do nothing in response to non-targets. Stimuli are presented simultaneously to a series of visual (e.g., animation of a flying airplane) and auditory (e.g., a dog’s bark) distractors. Distractors can appear singly or in pairs, in one or two modalities (auditory and visual). The cognitive load of the blocks differs: blocks 1 and 8 contain no distractors, blocks 2 and 3 contain visual distractors, blocks 4 and 5 include auditory distractors, and blocks 6 and 7 include visual and auditory distractors. During the second, fourth, and sixth levels, only one distractor is presented at a time. During the third, fifth, and seventh levels, two distractors are presented simultaneously. The Kids version of the test was administered to participants aged 6–12. The Teens & Adult version was administered to participants aged 13–17. The two versions are identical in terms of task and block structure. They differ in length (14.4 and 18.4 min, respectively) number of trials (53 and 59, respectively) and time of presentation of the stimuli (500, 1,000, or 3,000 and 500, 1,000, or 4,000 ms, respectively). Also, stimuli and distractors are different. In the Kids version, the target stimulus is a cartoon image of a child’s face. Non-target stimuli include five different images of animals. In the Teens & Adult version, the target stimulus is a cartoon image of a playing card with three red hearts, and the non-target stimuli are playing cards different from this (e.g., two red hearts and one diamond).

We used four indices: (i) *Omissions*: number of non-responses to the target during stimulus presentation or the void period; (ii) *Timing (T)*: number of correct responses performed *only* during stimulus presentation; (iii) *Impulsivity (I)*: number of commission errors, that is, the number of responses to non-target stimuli; and (iv) *Hyperactivity*: number of commission errors that were not coded as impulsive responses (e.g., multiple keypress in response to a target stimulus).

### Statistical analysis

All analyses were computed using the *Jamovi* software (The Jamovi Project, 2021). Descriptive analyses were performed on participants’ characteristics and MOXO d-CPT performance. Descriptive analysis revealed the violation of the assumption of homogeneity of variance (Shapiro–Wilk test) and the non-normal distribution of scores. For this reason, a series of generalized linear models (GLMs) were conducted to examine the effects of Sex and the covariate Age – treated as a continuous variable – as well as their interactions, on each MOXO d-CPT indices. Given the count nature of the data and the presence of overdispersion, a negative binomial distribution was used. Similarly, to investigate the impact of distractors on task performance, generalized linear mixed models (GLMMs) were fitted, also using a negative binomial distribution to appropriately model overdispersed count data. To reduce model complexity, we included four of the eight task conditions: block 1 (baseline), which did not involve distractors, and blocks 2, 4, and 6, which include visual, auditory, and combined distractors with low cognitive demand, respectively. To evaluate age- and sex-related differences in the impact of distractors, we tested the interactions between condition, Age (continuous variable as a covariate), and Sex. For both GLMs and GLMMs, *post hoc* analyses with Bonferroni correction were conducted for each significant effect. The model-building procedure for both GLMs and GLMMs included the following steps: first, unconditional models with linear, quadratic, and cubic age terms were estimated. The best-fitting model was selected based on the Akaike information criterion (AIC) and Bayesian information criterion (BIC). The selected unconditional model served as the basis for further model development. In all models, the age variable was mean-centered, and then the quadratic and cubic terms were computed from the centered age (Barr et al., 2013), to reduce multicollinearity and avoid convergence issues. Once the best-fitting growth model was identified, sex and condition were added in the models.

All the analysis were performed separately for the 6–12 and the 13–17 age groups because of the different structure of the two task versions.

## Results

A preliminary inspection of data suggests that the Omission, Hyperactivity, and Impulsivity scores were not normally distributed (Table 1 and Supplementary Figure 1).

### Age and sex related differences

A series of GLMs analyses were performed, including the task indices as dependent variable and age-centered and sex as predictors.

Among the GLM models tested for the 6–12 age group, the linear age term showed the best fit for all indices. Similarly, for the 13–17 age group, the linear age term was selected for all models. Notably, for this age group, linear and cubic models resulted as statistically equivalent for all indices, as the AIC and BIC values of the two models differ only marginally; however, given the non-relevant differences between models, visual inspection of the raw data suggested that the better fit may be due to random fluctuations in the sample. Therefore, the linear term was selected for all indices (see Supplementary Table 1 for models’ comparison).

#### Age group 6–12

**FIGURE 1:**
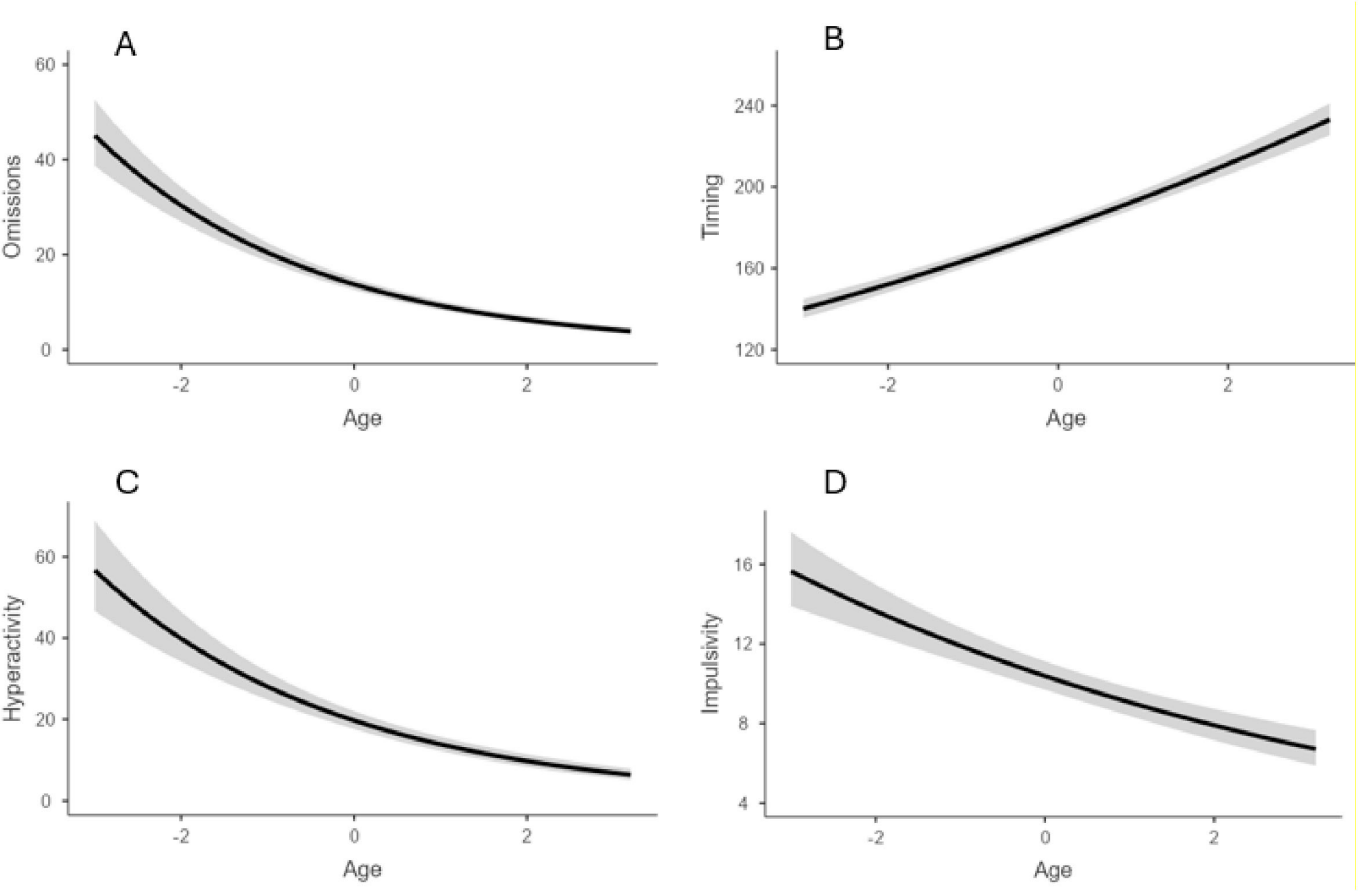
Impact of age for omission **(A)**, timing **(B)**, hyperactivity **(C)**, and impulsivity **(D)**. Age group 6–12. Age = age centered (*M* = 0, SD = 1).

**FIGURE 2:**
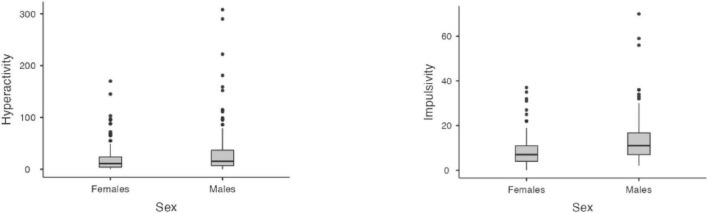
Sex differences in hyperactivity and impulsivity. Age group 6–12.

Considering Omissions and Timing, a significant effect of age emerged, with older age leading to a reduction of omissions (*z* = −17.4, *p* < 0.001) and an increment of the in-time responses (*z* = 17.83, *p* < 0.001). No effect of sex nor interaction age × sex emerged for the two indices (all *p* > 0.05). As for Hyperactivity and Impulsivity indices, an impact of age emerged (*z* = −12.57, *p* < 0.001 and *z* = −7.91, *p* < 0.001), with errors reducing by age. In addition, an effect of sex emerged for both indices (*z* = −3.23, *p* = 0.001 and (*z* = 6.10, *p* < 0.001), with males showing a higher rate of errors than females. Significant results are reported in Figures 1, 2.

#### Age group 13–17

**FIGURE 3:**
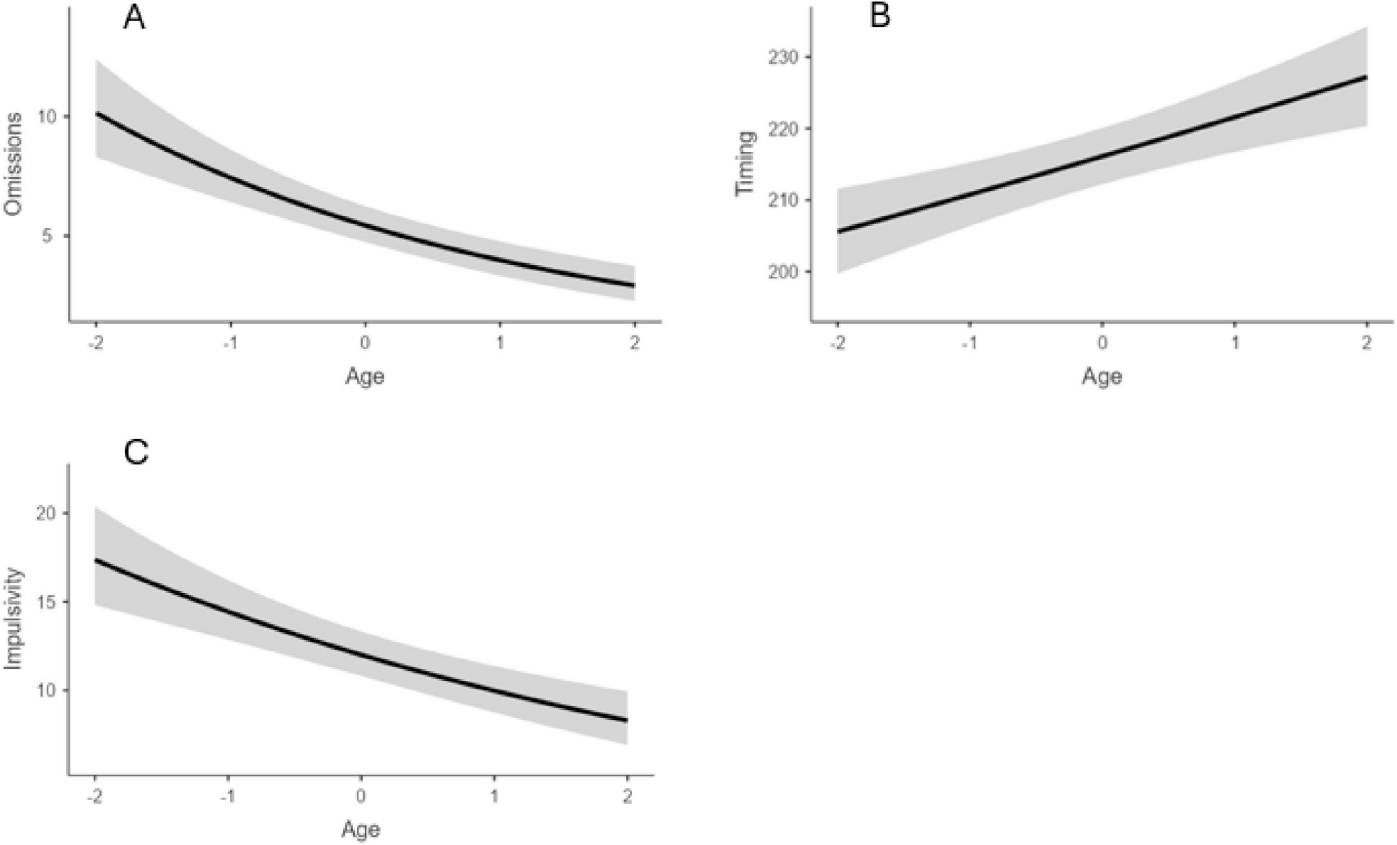
Impact of age for omission **(A)**, timing **(B)**, and impulsivity **(C)**. Age group 13–17. Age = age centered (*M* = 0, SD = 1).

**FIGURE 4:**
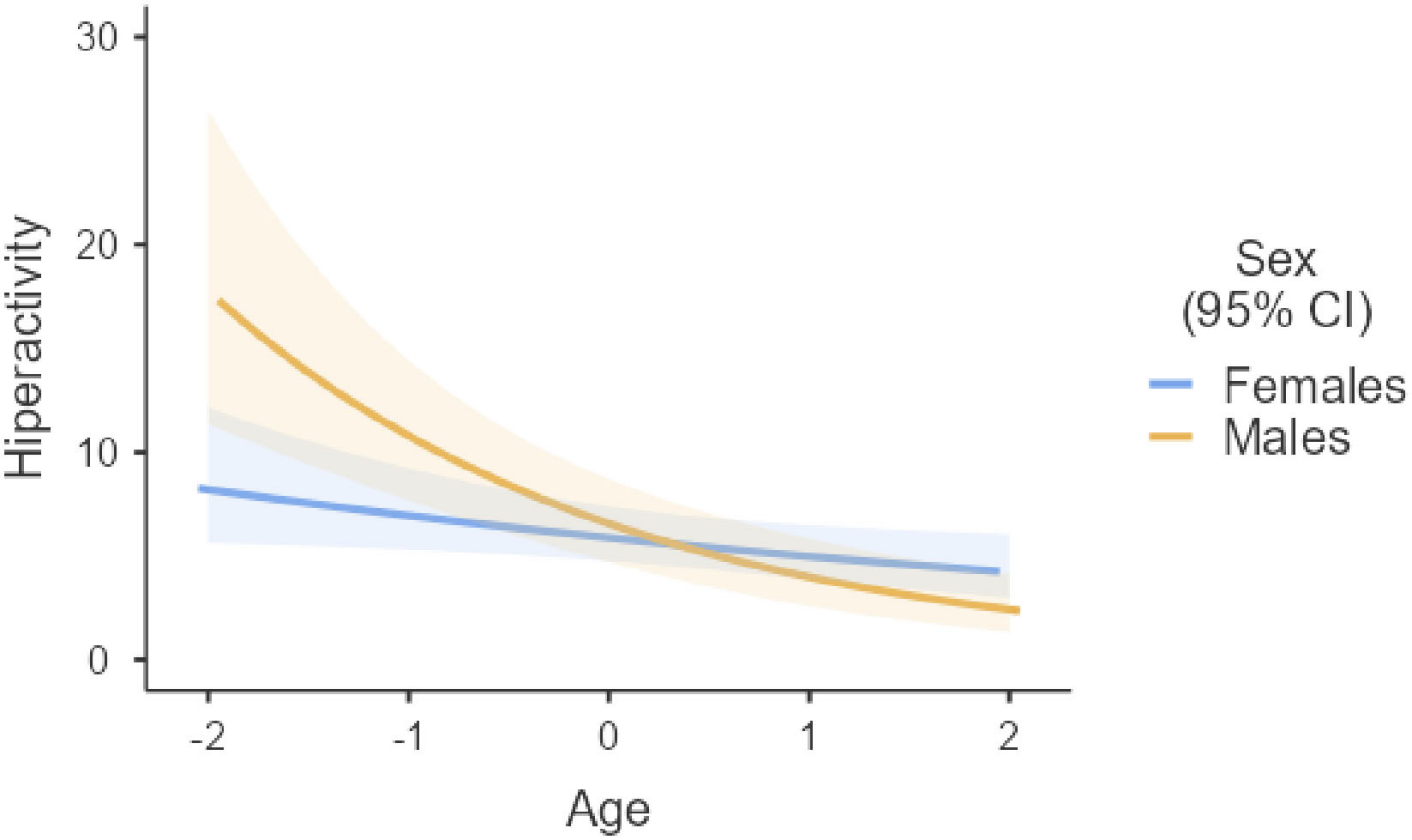
Age × sex interaction in hyperactivity. Age group 13-17. Age age centered (*M* = 0, SD = 1).

**FIGURE 5:**
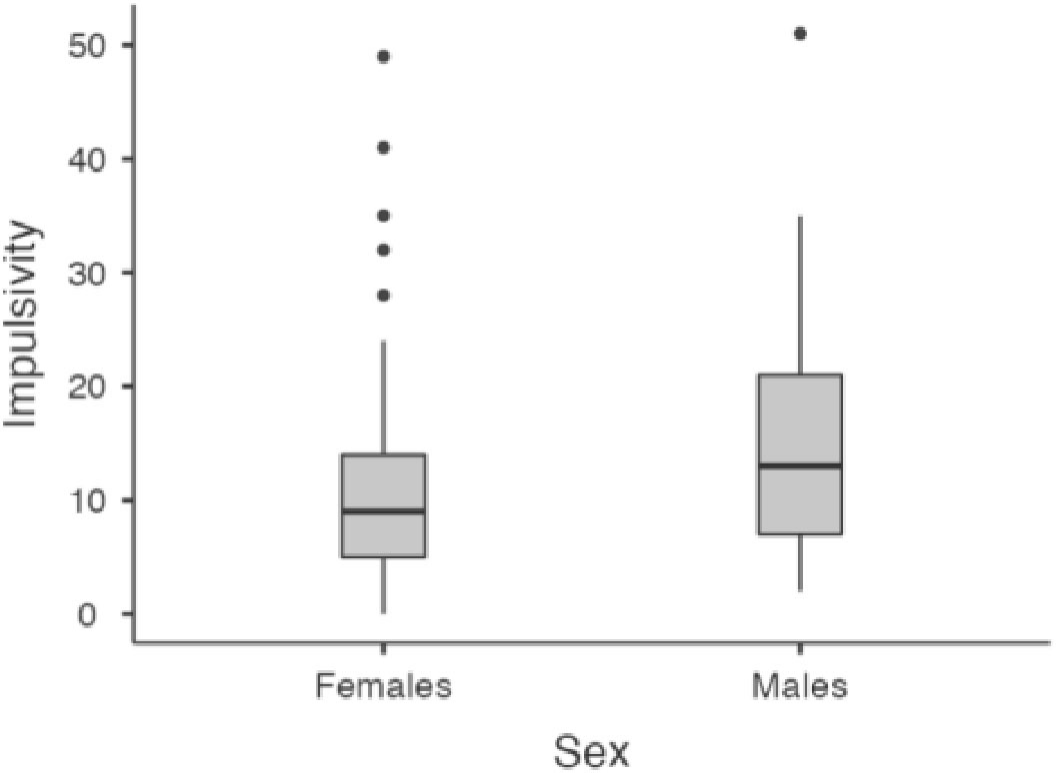
Sex differences in impulsivity. Age group 13–17.

Considering Omissions and Timing, a significant effect of age emerged, with older age leading to a reduction of omissions (*z* = −6.85, *p* < 0.001) and an increment of the in-time responses (*z* = 4.18, *p* < 0.001). No effect of sex nor interaction age × sex emerged for the two indices (all *p* > 0.05). As for Hyperactivity index, an impact of age emerged (*z* = −5.34, *p* < 0.001), with errors reducing by age. In addition, an interaction age × sex emerged (*p* = 0.008) with males showing a higher rate of errors at age 13, but a rapid reduction over time and a similar number of errors that females at age 17. Finally, considering the Impulsivity index, a significant impact of age emerged, with a reduction of errors over time (*z* = −5.38, *p* < 0.001). In addition, a significant impact of sex emerged (*p* = 0.038), with males showing the lowest performance (*z* = −2.06; *p* = 0.039). Significant results are reported in Figures 3–5.

### Impact of distractors

A series of GLMMs analyses were performed including the task indices as dependent variable, condition, age-centered and sex as fixed effects and subjects ID as a random effect. Among the GLMM models tested for the 6–12 age group, the linear age term provided the best fit for all the indices. Similarly, for the 13–17 age group, the linear age term was selected for all models. Notably, for the Timing and Impulsivity indices, the cubic term resulted in marginally lower AIC and BIC values; however, the model did not converge for the Timing index, and for the Impulsivity index, visual inspection of the raw data suggested that the better fit may be due to random fluctuations in the sample. Therefore, the linear term was retained for all indices to ensure consistency and model stability (see Supplementary Table 1 for models’ comparison).

#### Age group 6–12

**FIGURE 6:**
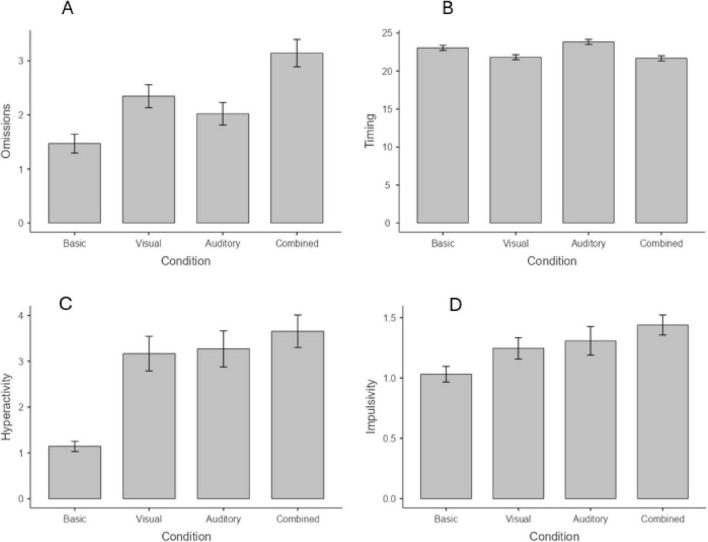
Impact of distractors on the task performance for omissions **(A)**, timing **(B)**, hyperactivity **(C)**, and impulsivity **(D)**. Age group 6–12.

The intraclass correlation coefficients (ICC) of the models were 0.37 for the Omission index, 0.51 for the Hyperactivity index, and 0.22 for the Impulsivity index, indicating that in these cases a significant portion of the variance in the dependent variable was explained by between-subject differences. The only exception emerges in the Timing index, where the ICC was 0.02, indicating that only a small portion of the variance (2%) was due to between-subject differences. Globally, these results justify the use of mixed models. As for the fixed effects, task condition was significant for all the four indices (all *p* < 0.001), indicating that the presence of distractors have an impact on the task performance. Interactions with age or sex were not significant for Omission, Timing, or Hyperactivity. Regarding Omissions (Figure 6A), *post-hoc* tests showed that the omission errors were significantly higher in all the three conditions with distractors compared to baseline, with the combined conditions having the greatest negative effect (*z* = −10.02, *p* < 0.001), followed by the visual (*z* = −5.94, *p* < 0.001) and the auditory condition (*z* = −3.22, *p* = 0.008). In addition, omission errors were significantly higher in the visual that in the auditory condition (*z* = 2.78, *p* = 0.033), while the combined condition determines a significant increment of the omission errors both comparing with the visual (*z* = −4.52, *p* < 0.001) and the auditory distractors (*z* = −7.13, *p* < 0.001).

For the Timing index (Figure 6B), visual and combined distractors lead to a reduction of in time responses (*z* = 3.01, *p* = 0.016 and *z* = 3.57, *p* = 0.002, respectively), in a similar way (condition 2 vs. 3, *p* = n.s.), while the auditory distractors do not impact the performance (*p* > 0.05). In addition, children show less timing in the visual condition than in the auditory condition (*z* = −5.08, *p* < 0.001) and in the combined that in the auditory condition (*z* = 5.62, *p* < 0.001).

Regarding Hyperactivity (Figure 6C), *post-hoc* tests showed that the errors were significantly higher in all the three conditions with distractors compared to the baseline (all *p* < 0.001, *z* = −9.11, −8.91, and −10.63, respectively), but no significant differences emerged between these three conditions (all *p* > 0.05).

**FIGURE 7:**
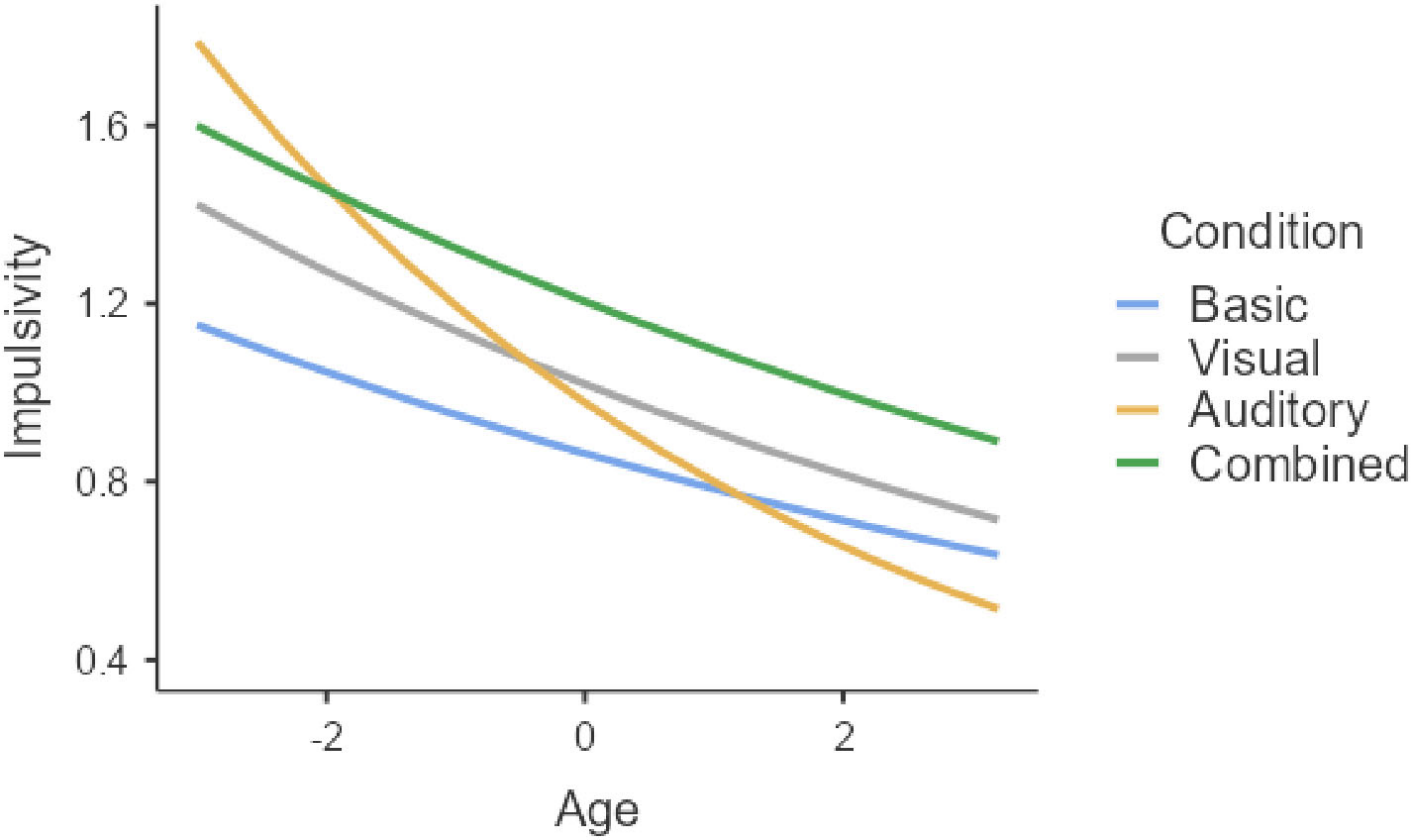
Interaction condition × age in Impulsivity index. Age group 6–12. Age = age centered (*M* = 0, SD = 1).

Finally, for the Impulsivity index (Figure 6D), only combined distractors impact the performance leading to an increment of the errors (*z* = −4.22, *p* < 0.001). In addition, a significant difference emerged between the auditory and combined conditions (*z* = −2.71, *p* = 0.040). An interaction condition × age emerged (*p* = 0.008), indicating that the impact of distractors decreased, with younger children being affected by all visual, auditory, and combined distractors (all *p* < 0.05), while older children being affected only by combined distractors (*p* = 0.006; Figure 7). The interaction further suggests that the detrimental impact of auditory distractors declines sharply with age, whereas the effects observed in the other conditions exhibit a more gradual and consistent decrease across the age span.

#### Age group 13–17

**FIGURE 8:**
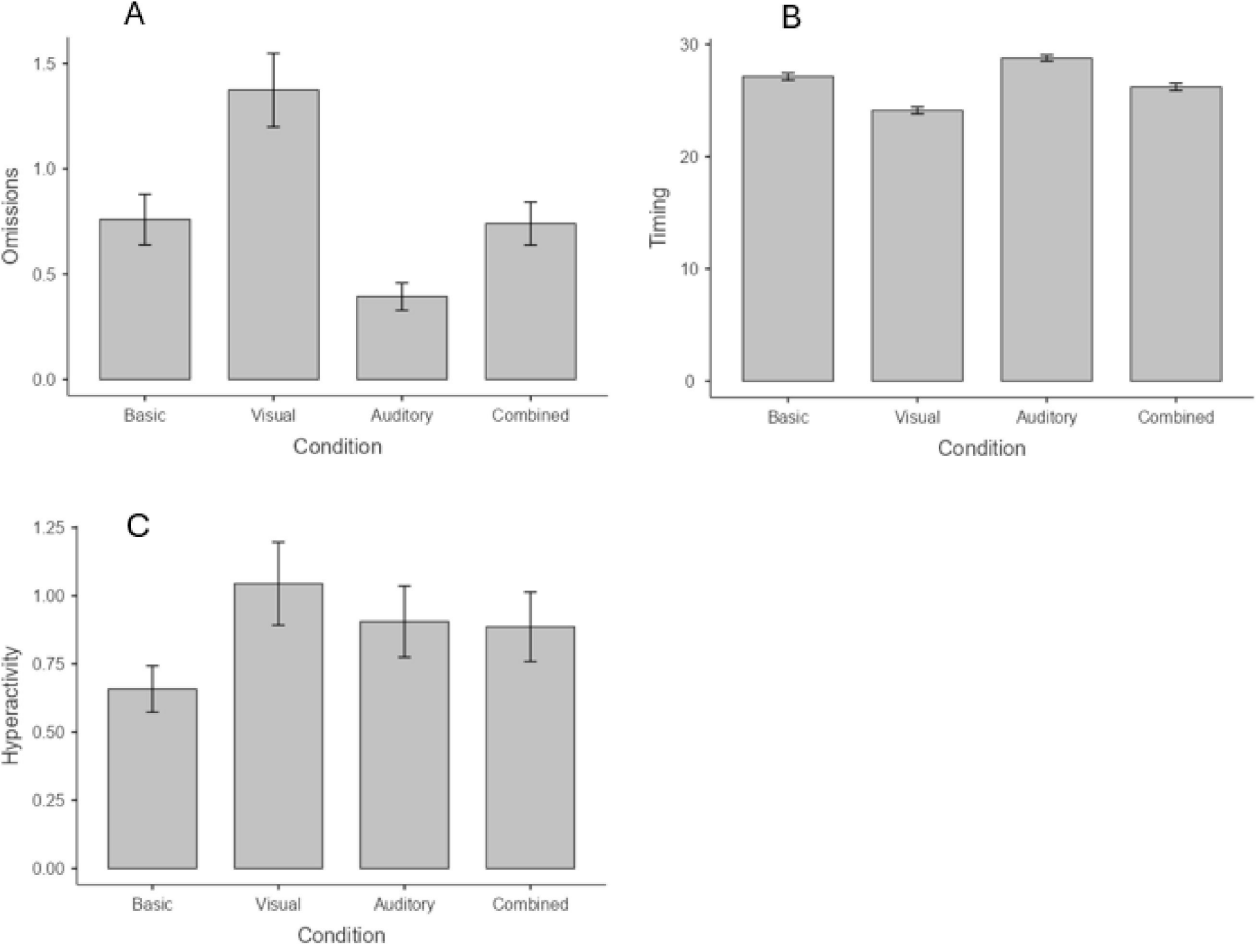
Impact of distractors on the task performance for omissions **(A)**, timing **(B)**, and hyperactivity **(C)**. Age group 13–17.

**FIGURE 9:**
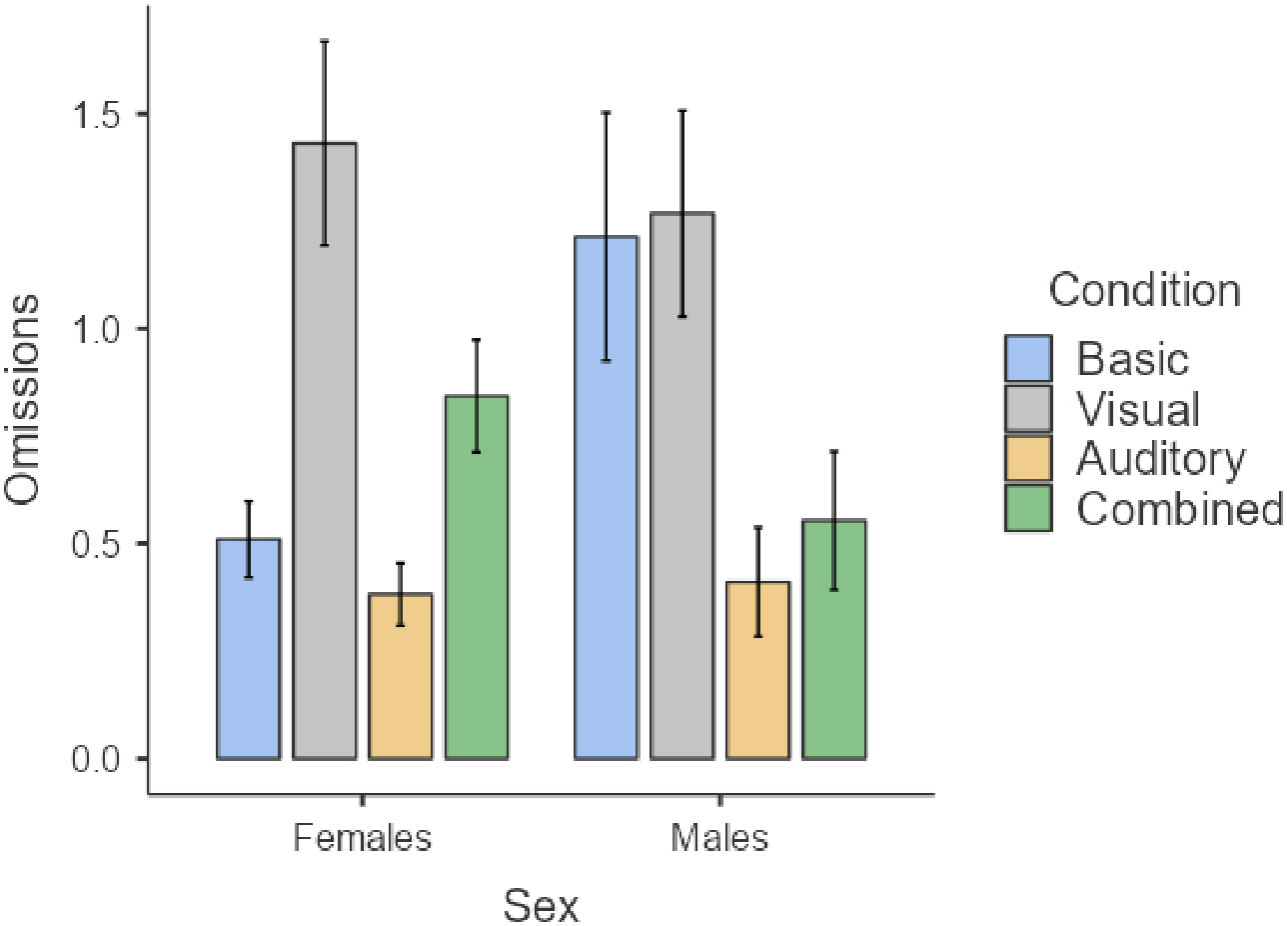
Impact of distractors in males and females in the Omission index. Age group 13–17.

The ICC of the models were 0.42 for the Omission index, 0.56 for the Hyperactivity index and 0.22 for the Impulsivity index, indicating that in these cases a significant portion of the variance in the dependent variable was explained by between-subject differences. The only exception emerges in the Timing index, where the ICC was 0.00, indicating that only a small portion of the variance (2%) was due to between-subject differences. Globally, these results justify the use of mixed models. As for the fixed effects, task conditions were significant for Omissions, Timing, and Hyperactivity (all *p* < 0.001), but not for Impulsivity (*p* > 0.05). For the Omission index, an interactions condition × sex emerged (*p* < 0.001). Regarding Omissions (Figure 8A), *post-hoc* tests showed that visual distractors result in poorer performance (*z* = −3.66, *p* = 0.002), whereas auditory distractors appear to enhance it (*z* = 3.45, *p* = 0.003). The combined condition has no impact on the performance (*p* > 0.05). That is, visual distractors lead to a poorer performance compared to both auditory (*z* = 6.65, *p* < 0.001) and combined (*z* = 4.86, *p* < 0.001) conditions, while no differences appear between auditory and combined distractors (*p* > 0.05). As for the interaction with sex, *post hoc* analysis revealed that for females, visual distractors increase omissions compared to the baseline (*p* < 0.001), while no differences emerged between the baseline and either the auditory or the combined condition. However, in this case, a non-significant trend toward an increment in omission errors was observed (all *p* = n.s.). For males, who exhibited more omissions than females at baseline, a different trend emerged: their performance remained stable between the baseline and visual distractor conditions, with a significant reduction in omission errors in both the auditory (*p* = 0.009) and combined (*p* = 0.014) conditions. In addition, both females and males showed a higher number of omission errors in the visual distractor condition compared to the auditory (*p* < 0.001 for females; *p* = 0.002 for males) and combined conditions (*p* = 0.015 for females; *p* = 0.003 for males). The increment of omission errors between auditory and combined distractors is significant only for females (*p* = 0.029; Figure 9). For the Timing index (Figure 8B), visual distractors lead to a reduction of in-time responses compared to the baseline (*z* = 4.56, *p* < 0.001), while auditory distractors improve the performance (*z* = −3.04, *p* = 0.014) and combined distractors do not impact (*p* > 0.05). In addition, adolescents showed lower on-time responses in the visual condition compared to both the auditory (*z* = −7.56, *p* < 0.001) and the combined conditions (*z* = −3.67, *p* = 0.001). Moreover, performance in the combined condition was significantly lower than in the auditory condition (*z* = 3.91, *p* < 0.001).

Regarding Hyperactivity index (Figure 8C), *post-hoc* tests showed that the errors were significantly higher in the visual distractors condition compared to the baseline (*z* = −2.73, *p* = 0.038). No other significant differences emerged. Finally, for the Impulsivity index, no significant main effect of the condition emerged, nor interaction with age or sex.

## Discussion

This study investigated age and sex-related differences in sustained attention and inhibitory control and the impact of distractors from mid-childhood to mid-adolescence. No previous studies investigated inhibitory control in adolescents, nor sex differences using a behavioral measure that provides separate scores for sustained attention, hyperactivity, and impulsivity and which in addition to classical CPTs also requires handling interference of distractors. Filling this gap can provide not only theoretical information on executive function development but also informs-tailored interventions and educational strategies during a developmental period of heightened vulnerability.

As for age related differences, in disagreement with the literature reported above, this study did not support any non-linear age-related trajectories of change in sustained attention and inhibitory control. By contrast, the results support the finding showing that sustained attention and inhibitory control develop over the period considered in a linear way (Bezdjian et al., 2014; Leon-Carrion et al., 2004). Even if this result is not in line with our hypothesis, it is important to note that previous studies using CPT tasks considered age as a categorical measure, comparing different age groups with the others, while in our study we preferred to consider age as a continuous variable. Our findings are consistent with prior research indicating linear developmental improvements in tasks measuring sustained attention and inhibition (e.g., Leon-Carrion et al., 2004). To note, in some cases our results do not reject the possibility of non-linear age effects, but non-linear models did not significantly improve model fit compared to the linear ones. Thus, a linear association between age and performance was retained for parsimony and interpretability.

Another interesting results relate to the differentiation of the Impulsivity and Hyperactivity indexes. While most inhibitory measures provide a single measure for these aspects, our results show that their developmental trajectories are different, with hyperactivity showing an earlier reduction compared to impulsivity.

Considering how sex influence the age-related differences, our results are in line with the literature, showing no sex differences in sustained attention, while males showing higher levels of Hyperactivity and Impulsivity than females across ages. Moreover, the significant interaction between age and sex for the Hyperactivity score indicates that boys become less impulsive from early to middle adolescence, reaching the hyperactivity level of girls who present a stable level of this index from early adolescence.

Taken together, these results suggest that sustained attention develops from mid-childhood to mid-adolescence, as previously demonstrated (Betts et al., 2006; Lewis et al., 2017; Yan et al., 2018), in a similar way for males and females (Brocki and Bohlin, 2004). By contrast, a different timing in inhibitory control development was observed based on sex, with males reaching later, in mid-adolescence, the level of performance already shown in early adolescence by females. An asynchrony in brain development (e.g., Kaczkurkin et al., 2019) may explain these different trajectories in inhibitory abilities, which consist of a more prolonged period of changes for boys and an early acquisition for girls (Usai, 2022). Interestingly, age- and sex-related differences in sustained attention and inhibitory control throughout mid-childhood to mid-adolescence appear to be characterized by different trajectories, confirming that they are separated and slightly correlated abilities (Boelema et al., 2014; Malagoli and Usai, 2018). In addition, some differences appear when different aspects of sustained attention and inhibitory control are considered, as the Impulsivity index remains more stable over time compared to the Hyperactivity one.

Considering the overall effect of distractors by age, our findings suggest a developmental trajectory of distractibility, with a general decrease in susceptibility to distractors as children age, culminating in the disappearance of distractor effects on the Impulsivity index during adolescence (Slobodin et al., 2018). Our findings provide new insights into the impact of distractor types on typically developing children and adolescents, highlighting both similarities and differences with prior research. Earlier studies, such as Uno et al. (2006), reported that typically developing children are primarily affected by visual distractors rather than noise. Cassuto et al. (2013), however, found no significant influence of auditory or visual distractors but observed performance decrements with combined distractors using the MOXO d-CPT. According to our hypothesis, the present study aligns with Cassuto et al. (2013), showing that combined distractors were the most impactful for children, followed by visual distractors, while auditory distractors had a smaller impact, also producing an enhancement on sustained attention in adolescents (Pelham et al., 2011; Slobodin et al., 2018). In addition, while in young children all distractors produce a decrement in the performance, in adolescents this emerge only for visual distractors, and only for sustained attention and hyperactivity. This is in line with previous studies showing that auditory stimuli may facilitate attentional performance through elevated arousal (Asutay and Västfjäll, 2017; Egeland et al., 2023; Söderlund et al., 2007). As for sex differences in the distractors’ impact, these emerges only in adolescents, where sustained attention emerged to be negatively affected by visual distractor for females, while in males an improvement in sustained attention emerges with auditory and combined distractors. These sex-related differences could be due to several factor. Although females are often found to have enhanced efficiency in visual processing (e.g., larger P1/P3 amplitudes; Jausovec and Jausovec, 2009), this heightened sensitivity may also increase their susceptibility to distraction. In this line, Steffensen et al. (2008) showed that among young adults aged 18–25, that females show an increased allocation of attentional resources to visual distracting stimuli than males. As for the male’s advantages with auditory distractors, previous studies showed that men but not females engage sensory gating mechanisms to maintain stable performance in a visual attention task with auditory distractors. This supports our observations of males improve their performance with auditory and combined distractors due to effective sensory filtering.

More recently, Rivella et al. (2024) explored distractor effects in children with ADHD, observing that visual distractors caused a pronounced decrease in sustained attention and impulse control (inhibitory control) in the ADHD group, a distinction that underscores differences between clinical and typically developing populations.

The present results demonstrate that distractors affect sustained attention and inhibitory control differently across age and sex, revealing developmental and sex-specific patterns not observable using traditional CPTs. This nuanced understanding contributes to the literature by providing evidence that distractors influence specific cognitive processes differently.

### Implications

These results are particularly relevant for the clinical setting and professionals working with children at risk of developing attention disorders, highlighting the importance of having distinct indices. Since hyperactivity tends to decrease at an earlier stage, it could serve as an early indicator to identify children at higher risk of exhibiting subsequent difficulties in sustained attention or inhibitory control. This allows the promotion of early support and interventions to avoid or reduce difficulties. However, further investigations are needed, as the results of this study do not allow for the verification of this hypothesis. In addition, results on distractors highlight how they can in some cases be a resource to maintain attention on a prolonged task and most importantly how males and female react differently to them, that is this should be taken into account in the school setting.

### Limitations

Some limitations of this study warrant mention. First, this is a cross-sectional investigation. As such, it cannot capture developmental changes over time, and longitudinal studies—tracking the same individuals across different developmental stages—may yield different and more nuanced findings. In particular, we were not able to assess the slope of the curve and evaluate any differences between the two capacities. Another limitation is due to the different versions of the task that do not allow one to catch the differences between the two age intervals covered by the Kids and the Teens & Adult versions, respectively. However, the presence of two versions of the task allows the adoption of a more sensitive and age-specific measure that can capture changes in the period considered (Berger et al., 2017).

## Conclusion

In conclusion, the results of this study contribute to the open debate about the development of different attentional processes and their associated sex-related differences, using a relatively new measure that, given the increased cognitive load due to distractors, appears to be particularly helpful in highlighting age- and sex-related differences. Additionally, the results support the potential utility of distinguishing between impulsivity and hyperactivity, as the latter index, typically not measured alone, is useful in identifying performance at risk as clinically relevant. They also highlight the impact of distractors on attention and inhibitory control, with important implications for both educational and clinical settings. Given the developmental sensitivity of MOXO in this normative sample of typically developing children, MOXO d-CPT has the potential to become a valuable source of information for the assessment of attention problems in children and young adults and the identification of children and young adults with attention disorders. Future research should address the diagnostic utility of the test.

## Data Availability

The datasets presented in this study can be found in online repositories. The names of the repository/repositories and accession number(s) can be found at: https://osf.io/nmj3w/?view_only=2d2e3f66ce4f475795e79bb2c84c56ac.
