# Supplementary material for: Sustained attention and inhibitory control: age and sex related difference in children and adolescents using a CPT with distracting events
Source: Front Psychol. 2025 Aug 20;16:1609537. doi: 10.3389/fpsyg.2025.1609537 (PMC12406707; doi:10.3389/fpsyg.2025.1609537)
Supplement: Supplementary file 1 [file Data_Sheet_1.pdf]

Figure S1. Descriptives statistics by indices and age.

Age 6-12 | **Age 13-17**

Omissions

Timing

Hyperactivity

Impulsivity

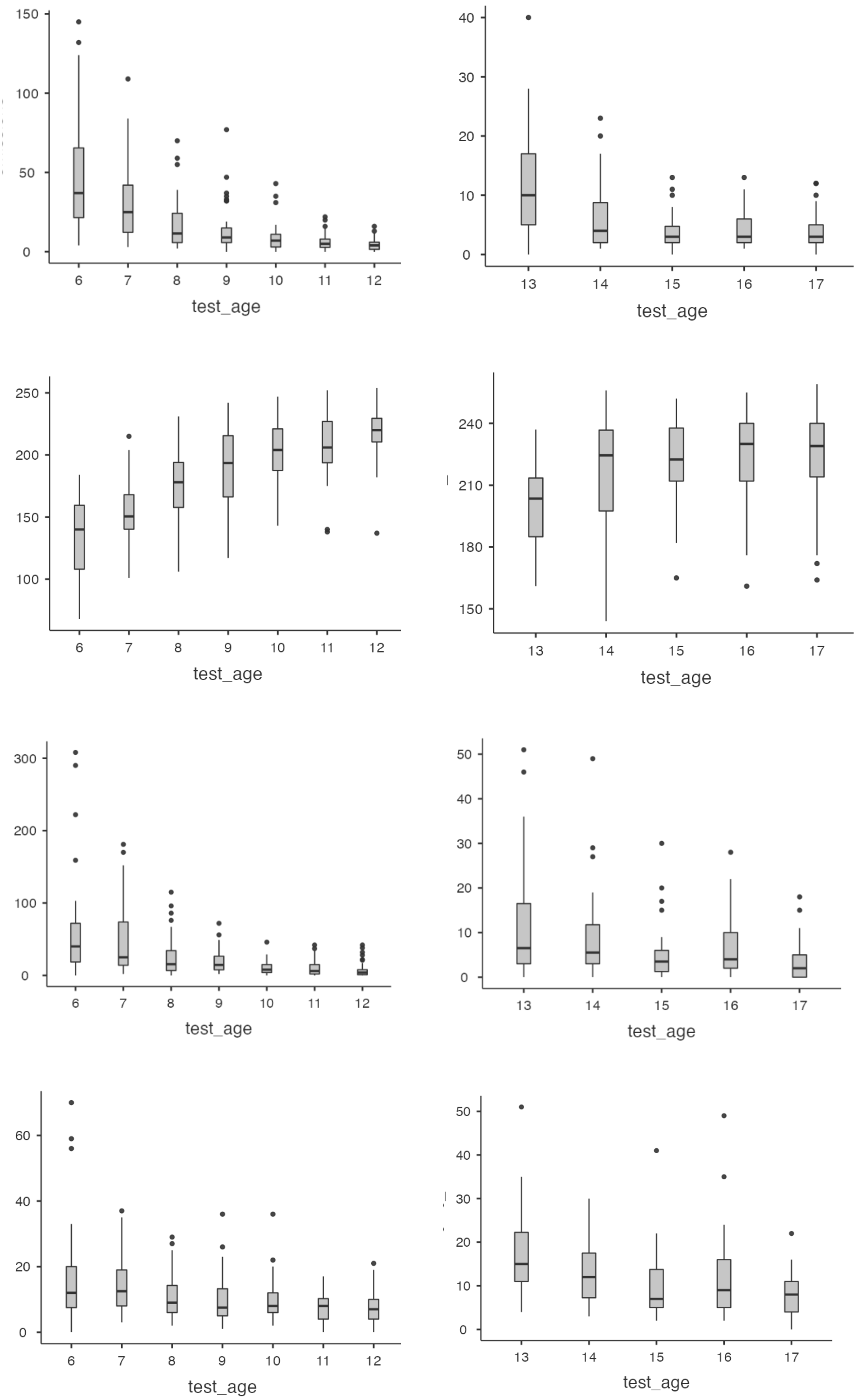

Table S1. GLMs and GLMMs unconditional models' comparison.

	AIC value for unconditional model					
	Age 6-12			Age 13-17		
	Linear age	Quadratic age	Cubic age	Linear age	Quadratic age	Cubic age
**GLMs**						
Omissions	**2324.27**	2520.72	2356.42	**874.54**	903.40	876.26
Timing	**3106.26**	3310.29	3138.52	**1455.47**	1467.14	1454.94
Hyperactivity	**2594.36**	2713.92	2625.75	**934.15**	958.21	934.63
Impulsivity	**2080.69**	2133.40	2087.27	**1053.36**	1082.13	1049.57
**GLMMs**						
Omissions	**3948.88**	4124.06	3972.96	**1458.61**	1458.61	1459.48
Timing	**6638.20**	6842.30	6671.98	**3633.77**	3641.01	3632.38
Hyperactivity	**4330.67**	4424.16	4348.97	**1428.92**	1450.98	1431.16
Impulsivity	**3277.55**	3312.49	3282.39	**1959.43**	1984.22	1957.66
